# A quantitative reference transcriptome for *Nematostella vectensis* early embryonic development: a pipeline for *de novo* assembly in emerging model systems

**DOI:** 10.1186/2041-9139-4-16

**Published:** 2013-06-03

**Authors:** Sarah Tulin, Derek Aguiar, Sorin Istrail, Joel Smith

**Affiliations:** 1 Eugene Bell Center for Regenerative Biology and Tissue Engineering, Marine Biological Laboratory, 7 MBL Street, Woods Hole, MA 02543, USA; 2 Department of Computer Science and Center for Computational Molecular Biology, Brown University, 115 Waterman Street, Box 1910, Providence, RI 02912, USA

**Keywords:** Transcriptome, Gene regulatory network, *Nematostella* embryonic development, Body plan evolution, Next-generation sequencing, Illumina HiSeq, Trinity, Oases, RNA-seq

## Abstract

**Background:**

The *de novo* assembly of transcriptomes from short shotgun sequences raises challenges due to random and non-random sequencing biases and inherent transcript complexity. We sought to define a pipeline for *de novo* transcriptome assembly to aid researchers working with emerging model systems where well annotated genome assemblies are not available as a reference. To detail this experimental and computational method, we used early embryos of the sea anemone, *Nematostella vectensis*, an emerging model system for studies of animal body plan evolution. We performed RNA-seq on embryos up to 24 h of development using Illumina HiSeq technology and evaluated independent *de novo* assembly methods. The resulting reads were assembled using either the Trinity assembler on all quality controlled reads or both the Velvet and Oases assemblers on reads passing a stringent digital normalization filter. A control set of mRNA standards from the National Institute of Standards and Technology (NIST) was included in our experimental pipeline to invest our transcriptome with quantitative information on absolute transcript levels and to provide additional quality control.

**Results:**

We generated >200 million paired-end reads from directional cDNA libraries representing well over 20 Gb of sequence. The Trinity assembler pipeline, including preliminary quality control steps, resulted in more than 86% of reads aligning with the reference transcriptome thus generated. Nevertheless, digital normalization combined with assembly by Velvet and Oases required far less computing power and decreased processing time while still mapping 82% of reads. We have made the raw sequencing reads and assembled transcriptome publically available.

**Conclusions:**

*Nematostella vectensis* was chosen for its strategic position in the tree of life for studies into the origins of the animal body plan, however, the challenge of reference-free transcriptome assembly is relevant to all systems for which well annotated gene models and independently verified genome assembly may not be available. To navigate this new territory, we have constructed a pipeline for library preparation and computational analysis for *de novo* transcriptome assembly. The gene models defined by this reference transcriptome define the set of genes transcribed in early *Nematostella* development and will provide a valuable dataset for further gene regulatory network investigations.

## Background

*Nematostella vectensis*, the starlet sea anemone, offers many advantages as a model system for the evolution of animal developmental programs. As an anthozoan cnidarian, it is strategically positioned as an outgroup to Bilateria [1-3] and is well situated to reveal the early steps in the evolution of the bilaterian body plan. Two of these evolutionary steps are likely to include the formation of a secondary body axis and a mesodermal germ layer which are both essential, defining characteristics of a bilaterian animal. Embryonic dorsal-ventral patterning and mesodermal development have been studied in many bilaterian models yet the origins of these significant body plan innovations are not well understood. Initial studies of gene expression in *Nematostella* and non-anthozoan cnidarians have revealed that genes important to bilaterian mesoderm specification are expressed in the endoderm of the sea anemone, and suggests that the bilaterian mesoderm may have originated from the endoderm of diploblastic ancestors [4-6]. Genes encoding factors involved in dorsal-ventral axis specification in Bilaterians are likewise asymmetrically expressed in *Nematostella*, indicating the possibility that a secondary axis was present in the Cnidarian-Bilaterian ancestor [7,8]. Defining the mechanisms controlling *Nematostella* development will help address these questions about the early evolutionary steps that led to bilaterian body plans with three germ layers and bilateral symmetry.

Gene regulatory networks (GRN) provide predictive models of gene regulation, as in the several examples that now exist for normal animal development (for example, *Drosophila*[9], sea urchin [10,11], ascidians [12], chick [13], and zebrafish [14]). To gain a comprehensive view of the control system, it is necessary to identify all genes whose products make up the regulatory network. This applies to our current research efforts but is also generally applicable to studies of virtually any regulatory system. Advanced sequencing platforms now allow us to do this through RNA-seq techniques. Yet, deep RNA-seq brings challenges in analysis reflecting the scale and complexity of transcriptomes, the primary problem being adequate assembly of RNA-seq reads in order to define a reference set of gene models [15-17]. Transcriptome assembly can be achieved using a reference-based strategy, a *de novo* strategy or a combination of the two. The main drawback to using a genome reference for assembly is that it relies on the quality of the reference genome being used [18]. This is a particular problem for emerging model systems with recently completed genomes because misassemblies, poor annotation and large gaps in coverage plague the genome assemblies of all but a few of the major model systems [19]. There is also a challenge in assigning reads that align equally well to multiple places in the genome. The aligner must decide to either exclude these reads which can result in gaps or to choose which alignments to retain which could lead to wrong assignments or predictions of a transcript in a region that has no transcription.

A comprehensive GRN for early embryonic development in *Nematostella* will enable researchers to investigate the extent to which the bilaterian regulatory toolkit is present in this representative cnidarian, down to the level of precise signaling systems and transcription factor *cis*-regulatory interactions. By harnessing the power of high-throughput sequencing and perturbation techniques, we aim to build the sea anemone GRN in an unbiased and efficient manner that will serve as a GRN construction pipeline for other model systems to follow.

The current *Nematostella* genome assemblies [20,21] fall into the category of young genome models that are still incomplete and contain gaps thus making the reference-based method alone insufficient for our needs. Taking these and all of the above complications into account and considering our goal to define an experimental and computational pipeline for emerging model systems, we elected to use the *de novo* assembly approach. This approach will be especially useful for evo-devo researchers aiming to harness the power of next-generation sequencing to bring their research into the genomics era; a trend already underway, for example *Parhyale*[22], *Oncopeltus*[23], sponge [24], and sea urchin [15].

The scale of reads, random and non-random sequencing errors, and inherent transcript complexity due to alternate transcription start sites or splice junctions all pose challenges for *de novo* transcriptome assembly. Indeed, the scale of the problem is only set to increase with the expanding capacity for transcriptome sequencing from advances in next-generation sequencing (NGS) platforms. In the last few years several assembly algorithms have been released to meet these challenges: Trans-ABySS [25], SOAPdenovo [26], Velvet/Oases [27,28], and Trinity [29]. The millions of short reads produced from NGS platforms result in millions of overlapping sequences. Short-read *de novo* assemblers exploit these overlaps to reconstruct the original transcripts by using the de Bruijn graph data structure, which encodes overlapping *k*-mers as adjacent vertices. Assembly algorithms then compute paths through the de Bruijn graph that correspond to valid assemblies of the sequence reads.

The Trinity assembler breaks the sequence data into many de Bruijn graphs in order to capture transcript complexity resulting from alternative splicing, gene duplications or allelic variation [29]. Trinity consists of three modules called Inchworm, Chrysalis, and Butterfly. Inchworm assembles the RNA-seq reads into transcripts and reports only the unique portions of alternate transcripts. Chrysalis clusters the Inchworm contigs so that each cluster represents all known transcripts variants for each gene or related genes and then constructs De Bruijn graphs for each cluster. All reads are segregated to one of these separate graphs. Butterfly then processes these separate graphs in parallel by tracing a path through each one and reports full length transcripts separately for alternate splice forms and paralogs. The Oases assembler uploads a preliminary assembly created by Velvet, which was originally designed for genome assembly. Oases corrects this assembly using a range of *k-*mers to create separate assemblies, which are then combined into one. The longer *k*-mers perform better on high expression transcripts and the shorter *k-*mers have an advantage on low expression transcripts [28]. While the multiple *k*-mer approach has been found to result in an increase of longer transcripts, it can also lead to an accumulation of incorrect assemblies or artificially fused transcripts [30].

In this study we designed a next-generation sequencing and analysis pipeline to produce a minimally biased and quantitative reference transcriptome. The resulting transcriptome represents the first 24 h of *Nematostella* development and will be the basis for further gene regulatory network studies. The experimental and computational pipeline will be used by us and others to produce transcriptomes for other model systems, particularly those evo-devo models that do not yet have an annotated genome but would benefit from an in depth molecular analysis.

## Methods

### Library prep

*Nematostella vectensis* adults following normal culture at 18°C were spawned with a 9-h cycle of light at 25°C in an incubator. Male and female spawning adults were in separate bowls and egg sacs were removed to a fresh bowl and fertilized with sperm from male bowls for 10 minutes. The egg sacs were then dejellied with a 4% cysteine solution (pH 7.4) in 50% filtered sea water (FSW) for 8 minutes and rinsed five times with 50% FSW. All embryo processing was performed in an 18°C room and the embryos were cultured from the time of fertilization for 0, 6, 12, 18 or 24 h (five timepoints). An additional 24-h sample was prepared in the same way from a separate spawning event. Cultured embryos were transferred to an eppendorf tube, allowed to settle, gently spun to a pellet and the supernatant removed, approximately 600 embryos per sample. The embryo pellet was immediately immersed in 100 μl of lysis buffer from the Invitrogen mRNA DIRECT kit (Invitrogen, Life Technologies, Grand Island, NY, USA) and homogenized with a Kontes Pellet Pestle (distributed by Thermo Fisher Scientific, Pittsburgh, PA, USA) attached to a 12 V/700 rpm drill. Another 100 μl of lysis buffer was used to rinse the Kontes Pellet Pestle tip and collected in the same tube. Samples were then stored at −80°C until all timepoints had been collected.

To thawed lysates, a third aliquot of 100 μl of lysis buffer was added and then the normal protocol for the Invitrogen mRNA DIRECT kit was followed using 50 μl Dynabeads per sample and low adhesion microcentrifuge tubes, following the manufacturer’s recommendations. The mRNA yields were between 108 ng and 344 ng total per sample. The mRNA was used as starting material for the ScriptSeq V.1 kit from Epicentre (Epicentre Biotechnologies, Madison, WI, USA). A total of 9.0 μl of mRNA corresponding to between 74 ng to 233 ng per sample was combined with 1.0 μl of a 1:10 dilution of External RNA Controls Consortium (ERCC) spike-in control RNA for the first reaction (available from Invitrogen/Life Technologies). The protocol was followed exactly, using 12 cycles total of PCR in the amplification step with Phusion High Fidelity polymerase (available from Therm Scientific) and barcoded Illumina-compatible primers 1 to 6 from Epicentre.

The libraries were size selected with a 2% Pippin prep gel (from Sage Science, Beverly, MA, USA) for 450 bp and checked on a Agilent 2100 Bioanalyzer with a high sensitivity DNA chip (from Agilent Technologies, Santa Clara, CA, USA) and then by qPCR. The samples were combined and run on a single lane of the Illumina High Seq 1000 with version III chemistry with 200 cycles of paired end sequencing plus indexing reads. All raw read files are available on the Woods Hole Data Archive at http://hdl.handle.net/1912/5613, DOI [DOI:http://10.1575/1912/5613].

### Computational methods

#### Quality control

Quality control was implemented using a combination of Bowtie2 (version 2.0.0-beta6), Basic Local Alignment Search Tool (BLAST), btrim (build date 9 September 2011), and the FASTX-Toolkit. First, we computed overrepresented *k*-mers in the raw sequence data and ran BLAST against the set of non-redundant (nr) sequences from the National Center for Biotechnology Information (NCBI). The top several BLAST hits were analyzed for homology with ribosomal or mitochondrial RNA. Using the BLAST data, we constructed a catalog of rRNA and mtRNA sequences which could serve as a reference set to filter the overly abundant non-protein coding RNA prior to *de novo* transcriptome assembly. A Bowtie2 index was built from the rRNA and mtRNA sequences of *Montastraea franksi*, *Savalia savaglia*, *Actiniaria*, *Nematostella vectensis*, and *Clathrina*. Sequence reads successfully aligning by Bowtie2 to this set were removed. The overrepresented *k*-mers also contained adapter sequences that remained in the sequence. We retrieved the exact Illumina adapter sequences and used the software tool btrim to clip adapters.

Sequence reads demonstrating low complexity (containing only one or two unique bases) are likely due to technical artifacts and were removed. Next, the GC content distribution was computed for the set of all reads. GC content biases in the first 13 bases of Illumina RNA-seq data are known to exist due to random hexamer priming [31]. This bias may cause an imbalance in read coverage and persist through the assembly process, which can affect the quality of assembly and quantification levels. Because we had extremely high sequence coverage, we removed the bias by simply trimming the start of the reads. Using the FASTX-Toolkit, we removed the initial 13 bases from the reads at each timepoint. Finally, btrim was also used to adaptively trim low quality bases from the end of the read. Adaptive trimming is performed by sliding a window of 5 bp from the end of the read to the start, removing bases and shifting the sliding window by 1 base if the average quality score is less than 30 until the average quality score is at least 30.

#### Digital normalization, Velvet and Oases

Digital normalization is a method to reduce the total number of reads to be assembled, thereby also reducing the computing power and time required for assembly. It preferentially removes high abundance reads but retains read complexity in order to remove errors but preserve low abundance transcripts prior to assembly. All links to digital normalization software are available electronically through http://ged.msu.edu/papers/2012-diginorm/. Raw paired-end read files were first interleaved into pairs using a python script, available at http://github.com/ged-lab/khmer/tree/2012-paper-diginorm/sandbox. Then, three rounds of digital normalization were applied to remove overabundant and erroneous reads. These depend on the khmer software package, available at http://github.com/ged-lab/khmer/. The khmer software also relies on the screed package for loading sequences, available at http://github.com/ged-lab/screed/ (khmer and screed are ©2010 Michigan State University, and are free software available for distribution, modification, redistribution under the BSD license). The digital normalized files were assembled with Velvet (version 1.2.03) and Oases (version 0.2.06). The details of the execution commands are available in Additional file 1. The most current recommendations for use of digital normalization for *de novo* transcriptome assembly recommend using only one round of normalization instead of three. Fewer low abundance transcripts may be lost by foregoing further rounds of digital normalization at the expense of increased computing time and power to assemble the greater number of remaining reads.

#### Trinity assembly and quantification

**Figure 1:**
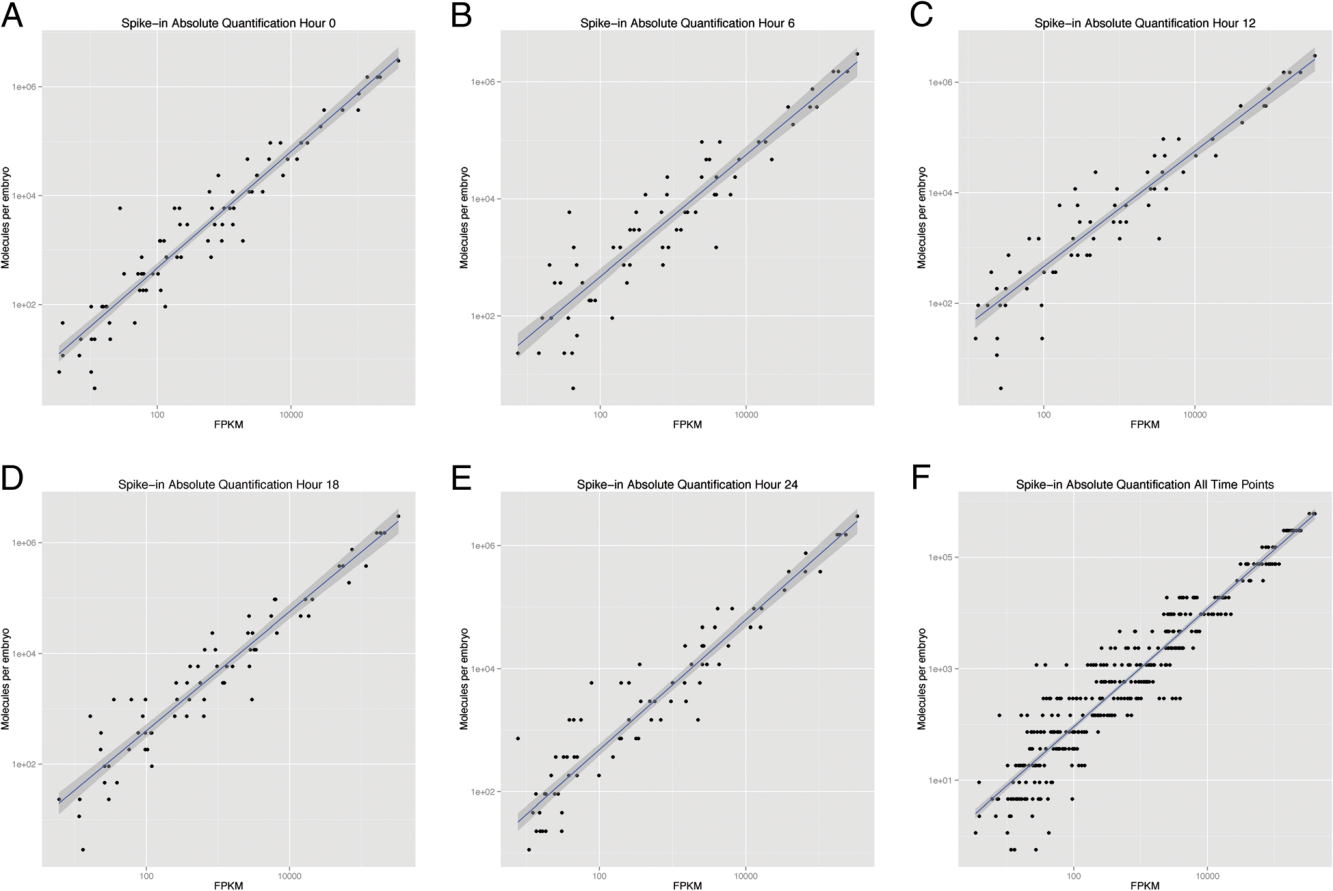
**Standard curves for RNA spike-in control standards.** RNA spike-in control standard curves for (**A**) 0 h (**B**) 6 h (**C**) 12 h (**D**) 18 h (**E**) 24 h and (**F**) all timepoints. The x-axis shows the fragments per kilobase of exon per million fragments mapped (FPKM) values (reads) and the y-axis shows the known concentrations of each molecule, in molecules per embryo. The blue line and grey shadow represent the best-fit line using robust linear regressions and standard error, respectively.

The 20 August 2011 release of the Trinity pipeline was run on the reads remaining after quality control (http://trinityrnaseq.sourceforge.net/). We ran Trinity with the options to use eight CPU cores and the RF library type to reflect the directionality of the sequence reads (full execution commands are given in Additional file 2). The assembled transcriptome is available on the Woods Hole Data Archive at: http://hdl.handle.net/1912/5613, [DOI:http://10.1575/1912/5613]. Bowtie was then used to align the post quality control sequence reads to the transcriptome assembly and the ERCC spike-in control sequences. We then computed (1) the set of concordant paired-end mapped sequence pairs and (2) the set of all mapped sequences for both the transcriptome and the ERCC controls. Fragments per kilobase of exon per million fragments mapped (FPKM) values were computed for the transcriptome assembly transcripts and the ERCC controls using RSEM (version 1.2.0). To quantify the expression of transcripts in terms of molecules we computed the dose–response curve by plotting FPKM values versus the known concentration of each ERCC spike-in for each timepoint. A set of ordinary least squares (Additional file 3) and robust linear regressions (Figure 1) [32] were computed, and we observed that the set of concordant mapped reads yielded higher R^2^ (Table 1) than the set of all mapped reads, and thus, we used the concordant mapped reads for downstream analyses. Using the fitted line, we inferred the number of molecules present for each Trinity assembled transcript, in each timepoint.

**Table 1:** Spike-in standard curve R values for the ordinary least squares regression

**Time**	**R**^**2**^
Hour 0	0.973
Hour 6	0.959
Hour 12	0.969
Hour 18	0.957
Hour 24	0.964
All hours	0.963

#### Blast2GO

To compute overexpressed GO terms in our transcriptome, we used BLASTx 2.2.26+, BLOSUM62 similarity matrix, Blast2GO database version August 2011, and pipeline B2G4Pipe version 2.3.5. The definition of each GO term is determined by the GO Consortium: http://www.geneontology.org/, and can be found using the EMBL-European Bioinformatics Institute QuickGO: http://www.ebi.ac.uk/QuickGO/, or the Gene Ontology Normal Usage Tracking System, GONUTS: http://gowiki.tamu.edu/wiki/index.php/Main_Page. Definitions for all GO terms presented in this paper can be found in Additional file 4.

## Results

### Library preparation and quality control of reads

**Figure 2:**
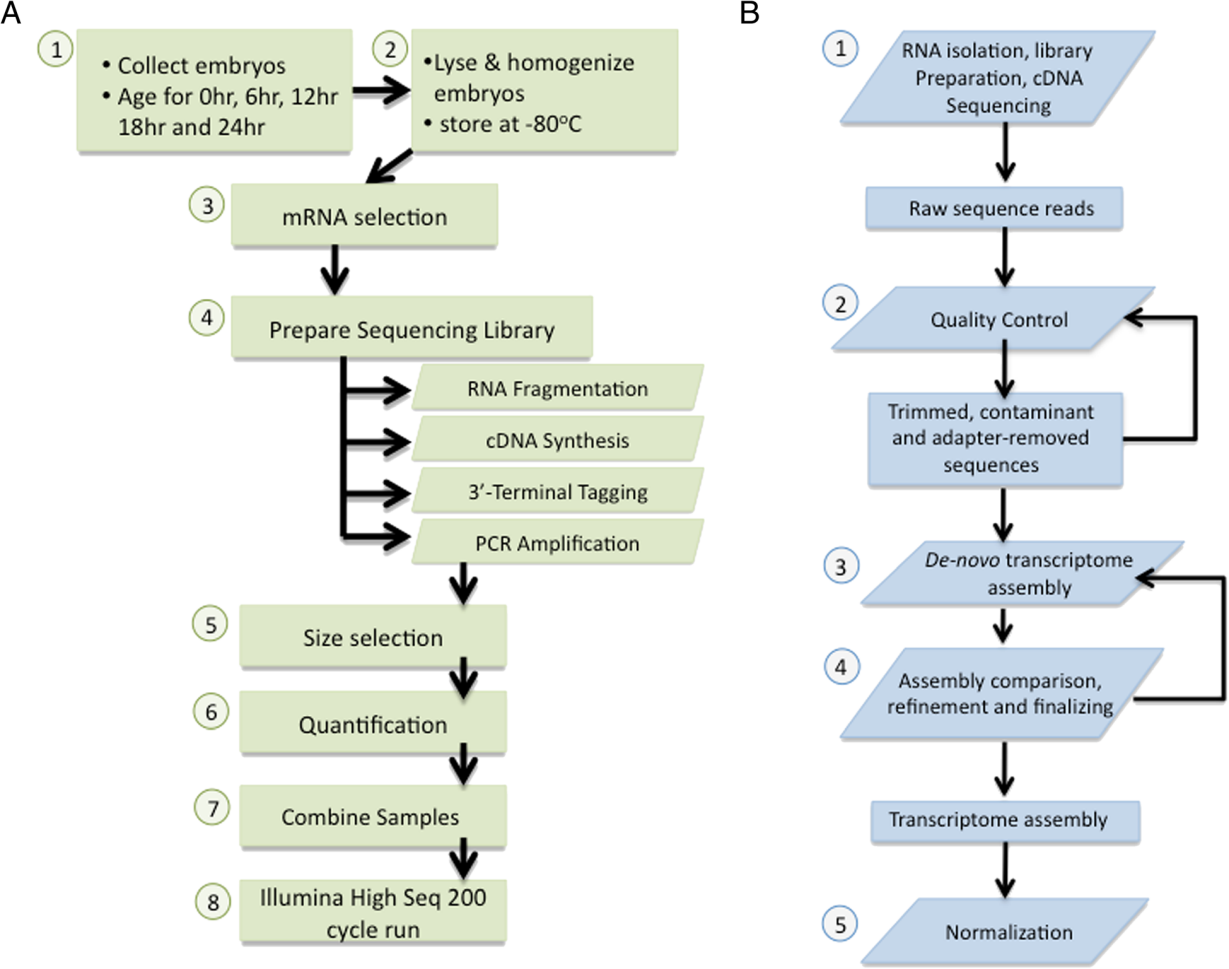
**Experimental and computational workflow.** (**A**) (1) Harvesting embryos after fertilization and subsequent aging all took place at 18°C. (2) After lysis and homogenization of embryos using Invitrogen lysis buffer and a pestle, lysates were stored at −80 degrees. (3) Protein-coding mRNA species were selected directly using the mRNA DIRECT kit from Invitrogen. (4) Sequencing libraries were prepared using the ScriptSeq kit from Epicentre/Illumina. The kit has four major steps: RNA fragmentation, cDNA synthesis, 3’-terminal tagging, and PCR amplification. Separate index tags were added to each timepoint during PCR amplification. (5) Libraries were size selection using a 2% pippin prep from Sage Science to 450 bp. (6) Quantification of libraries with Bioanalyzer and qPCR. (7) All samples were pooled into a single lane. (8) Sequencing was performed on an Illumina HiSeq 1000 with version 3 chemistry. (**B**) (1) Wet-lab experimental methods to isolate, prepare, and sequence the RNA result in raw reads. (2) Quality control removes adapter sequences, artifacts, ribosomal and mitochondrial contaminants; trims the GC-content bias; and adaptively trims the low quality sequence bases. (3) *De novo* transcriptome assembly using Trinity or Velvet-Oases. (4) The transcriptome assemblies are compared using a variety of metrics. (5) Reads are normalized prior to quantification and other downstream analyses. Reads for specific transcripts are normalized based on fragments per kilobase of exon per million fragments mapped (FPKM) and spike-in measurements.

In theory, RNA-seq can catalog all expressed transcripts as complete mRNA sequences. To determine the set of transcripts expressed from fertilization to gastrulation, we chose to sample five timepoints during the first 24 h of *Nematostella* development: 0, 6, 12, 18, and 24 h after fertilization (Figure 2A). First, 600 embryos per timepoint were immediately homogenized and lysed with lysis buffer from the Invitrogen mRNA DIRECT kit and stored at −80°C until all samples had been collected. Lysing immediately was important, as freezing the embryos as a pellet results in significant RNA degradation (Antje Fischer, Marine Biological Laboratory, Woods Hole, MA, USA, personal communication). Next, mRNA was positively selected with the Invitrogen mRNA DIRECT kit, which is a magnetic bead-based method. We tried an alternate mRNA enrichment method with total RNA extraction combined with negative selection for ribosomal RNAs, but the yields were too low from total RNA extraction with a Qiagen total RNA kit even for use with the low input version of the RiboZero kit from Epicentre. This is probably due to low RNA levels or difficult to extract RNA (not an uncommon problem in embryo systems) in *Nematostella* embryos, so for this step in the pipeline the alternate negative selection method may work better for species such as *Xenopus*, which have large amounts of RNA in their eggs. Our research group has more recently used this alternate method successfully for developing embryos of the slipper snail, *Crepidula fornicata*.

The polyA-RNA enriched sample was then processed with the Script Seq kit, version 1, from Epicentre. The main advantages of this kit are the resulting directional reads, short preparation time (4 h), and low input requirements (as low as 50 ng mRNA). The adapter-ligated libraries were then size selected for uniformity at 450 bp using a Pippin Prep gel electrophoresis apparatus from Sage Science and combined in one lane on an Illumina HiSeq 1000 to produce 2 × 100 bp paired-end reads.

The sequencing run produced 238.5 million total raw reads (1.19E + 08 pairs; Table 2), yielding far more than 20 GB of data. Quality control was then implemented in two phases (Figure 2B). The first phase removes adapter sequence contamination and ribosomal and mitochondrial RNA sequence. The second phase filters low complexity artifacts that may have resulted from technical failures during sequencing, removes low quality bases from the ends of reads, and trims the GC-content bias sequence bases from the start of the each read (see Methods section for more details). Both quality control phases may remove reads entirely or a subset of bases. If the length of a read is less than the *k*-mer length of the *de novo* assembler it cannot be used for assembly and is removed from the read set. These filters may therefore result in read fragments that have one of the paired reads removed while the other passes quality control. The raw sequence dataset contains 24 billion bases in 119 million paired directional sequence reads. After quality control phase 1 (QC phase 1), 71.7% of the bases and 70.3% of the paired sequence reads remained (Table 2). Phase 1 removed one of the pairs from 1.3 million fragments effectively introducing unpaired sequences into the read set. After QC phase 2, 59.1% of the original sequence bases remained and 67.5% of the original paired sequence reads remained. A total of 5.4 million unpaired sequences remained after QC phase 2.

**Table 2:** Quality control read attrition

**Timepoint**	**Raw paired end reads**	**QC step 1**	**%**	**QC step 2**	**%**
0 h	1.74E + 07	1.29E + 07	74.29	1.24E + 07	71.38
6 h	1.98E + 07	1.46E + 07	73.76	1.40E + 07	70.95
12 h	1.44E + 07	9.17E + 06	63.48	8.76E + 06	60.68
18 h	2.88E + 07	1.96E + 07	68.25	1.89E + 07	65.65
24 h-A	3.61E + 07	2.57E + 07	71.05	2.46E + 07	68.22
24 h-B	2.75E + 06	1.87E + 06	67.99	1.77E + 06	64.31
Total	1.19E + 08	8.39E + 07	70.31	8.05E + 07	67.51

### *De novo* assembly with Trinity assembler

**Figure 3:**
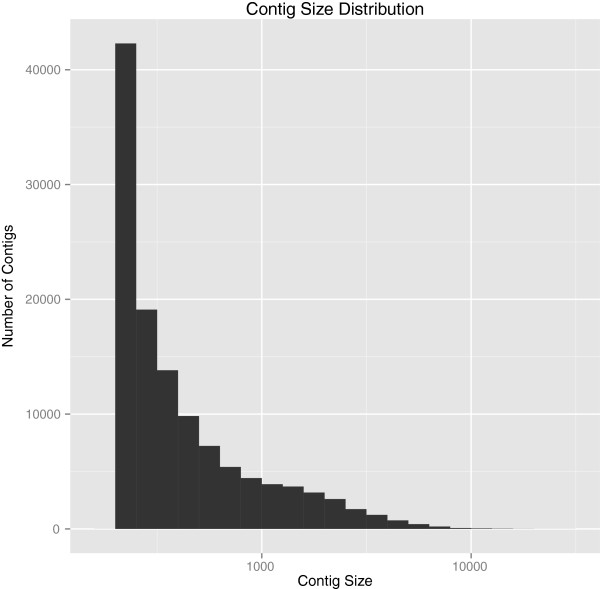
**Distribution of contig length for assembled reads.** Contig length distribution from Trinity-assembled reads. Assembled reads that passed quality control formed 119,911 contigs where 14.85% were more than 1,000 bp long.

Assembling a transcriptome from short reads is computationally challenging and several methods exist for assembly using an annotated genome as a reference, assembling the reads *de novo* without a genome reference, or a combination of the two. Due to the aforementioned difficulties with using the current *Nematostella* genome for assembly, we chose to compare two alternate pipelines which both use *de novo* assembly exclusively. The first uses the Trinity platform, which has been shown to recover more full-length transcripts across a range of levels at a sensitivity level comparable to assemblers that use a genome reference [33,34]. Additionally, Trinity can recognize alternate splice forms as belonging to the same gene and keep them together with the same prefix. Sequence reads that passed quality control were assembled into 119,911 contigs by Trinity (Table 3); 14.85% of assembled contigs were more than 1,000 bp long (Figure 3). The total alignment rate for the Trinity assembly was 85.90% (Table 4).

**Table 3:** No. of distinct transcripts passing filters

**Type**	**No.**
Total number of assembled transcripts	119,911
≥1 BLASTx hit (*Nematostella vectensis*), e <5e-5	104,438
≥1 BLASTx hit (non-redundant), e <5e-5	61,835
≥1 BLASTx hit (non-redundant) and 80% similarity	48,235
≥1 BLASTx hit (*Nematostella vectensis*) and 80% similarity	47,193
Molecules per embryo (MPE) >100	8,154
MPE >100, ≥1 BLASTx hit (non-redundant)	6,169
Transcript families from transcripts with MPE >100, ≥1 BLASTx hit (non-redundant)	4,055

**Table 4:** Trinity assembly compared to digital normalization/Velvet/Oases

	**Trinity assembly**	**Digital normalization, Oases-Velvet**
Total fragments	87,209,130 (100.00%)	87,209,130 (100.00%)
Aligned concordantly 0 times	23,101,349 (26.49%)	35,552,373 (40.77%)
Aligned concordantly 1 time	28,325,506 (32.48%)	30,552,963 (35.03%)
Of reads aligning concordantly or discordantly 0 times:		
Aligned discordantly 1 time	1,826,073 (7.90%)	7,539,150 (21.21%)
Total mates	42,550,552	56,026,446
Aligned 0 times	24,594,154 (57.80%)	31,100,316 (55.51%)
Aligned exactly 1 time	4,660,328 (10.95%)	11,909,789 (21.26%)
Aligned >1 times	13,296,070 (31.25%)	13,016,341 (23.23%)
Overall alignment rate	85.90%	82.17%

Determining adequate coverage of transcriptomes is more challenging than determining coverage of genomes because every transcript species (including splice variants or those using alternate transcription start sites) is present at a different level across a large range. Several studies have used an independent assembly of randomly selected subsets of their reads to compare the rate of new transcript discovery, determine the lower abundance limit of detection and compare the average length of isotigs. While analyzing the sea urchin embryonic transcriptome, Tu *et al*. compared a 20 M read subset with a 2 M read subset and a 0.2 M read subset, and found that 20 M reads were sufficient to reliably detect levels of transcripts at 400 molecules/embryo, which they estimate as the lower limit for proteins of developmental significance (such as transcription factors, which may be functionally relevant at levels as low as 10 copies of transcript per cell) [15]. In their analysis of the milkweed transcriptome, Ewen-Campen *et al*. created eight subsets of reads, assembled them separately and used BLASTx to compare gene discovery rates [23]. They found that the rate of new transcript discovery plateaued at 1.5 M reads, although the N50 isotig length continued to increase when using 2 M to 17 M read subsets. After 2 rounds of quality control filtering of our reads, we were left with 80,537,812 paired and 5,362,854 unpaired reads, a depth which has been shown to produce good sensitivity in these other systems for identifying all protein-coding transcripts expressed in the early embryo.

To restate, these previous studies indicate that with the volume of reads coming off the latest Illumina platforms (250 million to 400 million reads/lane), and only multiplexing 6 samples in a lane, we should be beyond the necessary coverage to represent all relevant regulatory transcripts. The best indication of the quality of our assembly is that we have been able to use it as a reference to map reads from more recent experiments in our lab at a median 93% rate (with 90% of samples mapping 90% of their reads to the Trinity-assembled reference).

### Digital normalization followed by Oases assembly

To evaluate more closely the quality of our *de novo* transcriptome assembly, we compared Trinity with an alternate strategy that combines a digital normalization step [17] with the assemblers Velvet [27] and Oases [28]. Digital normalization is a computational normalization method that preferentially removes high abundance reads but retains read complexity in order to remove errors and preserve low abundance transcripts prior to assembly. The quality controlled reads were assembled using Velvet and Oases and then mapped back to the resulting assembly (commands in Additional file 1). The main advantage of this method is it greatly decreases the computing power and time required to process millions of reads. We also tested the Amazon Elastic Cloud Computing Service (Amazon EC2), http://aws.amazon.com/ec2/, to perform the digital normalization and Velvet-Oases assembly. This method proves to be a great alternative to using a home institution’s core computers if the institution does not have sufficient computing power for running an assembler or if the computers are expensive to rent, slow or unreliable. Whereas the computation of Trinity required over 50 h and 100 GB of RAM in addition to the quality control steps, the pipeline using digital normalization, Velvet and Oases can all be run in a single day using an XL computer rented from Amazon. We found that this alternative assembly approach gave competitive results when mapping our *Nematostella vectensis* RNA-seq reads. The overall mapping success rate was 82.17% for digital normalization-Oases as compared to 85.90% for Trinity (Table 4).

### Quantification of Trinity-assembled transcriptome using known RNA ‘spike-ins’

**Figure 4:**
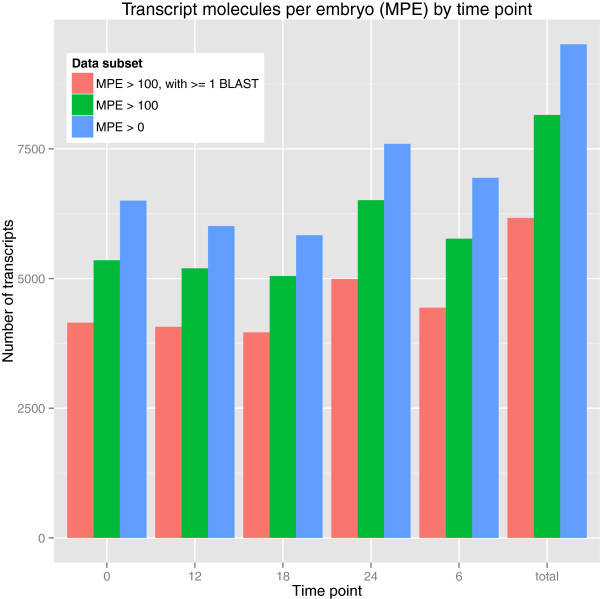
**Number of transcripts by timepoint.** Graph displaying the number of transcripts per timepoint above 3 thresholds: molecules per embryo (MPE) greater than 0 (blue bars), MPE greater than 100 (green bars), and MPE greater than 100 with at least 1 Basic Local Alignment Search Tool (BLAST) hit (red bars).

A key component of our *de novo* transcriptome pipeline is a method to obtain absolute quantitative information for each transcript using external standards or ‘spike-ins’, that is, control RNAs of known concentration. Before absolute quantification of inferred transcripts can be performed, the dynamic range and transcript detection limits must be evaluated using spike-ins. For this we employed the ERCC RNA spike-in set as recommended by the National Institute of Standards and Technology (NIST) [35-37]. First, we computed the properly mapped read alignments from quality controlled sequence read pairs to the ERCC spike-in reference sequences for each embryological timepoint. Quantification of spike-in transcripts was then performed using RSEM [38]. Read alignments that were not concordant with directionality constraints were not considered for quantification. We then compared the known concentration of each ERCC spike-in transcript to the RSEM calculated FPKM values. The dose–response curve for each timepoint containing the known concentrations and FPKM values for the spike-ins were plotted. We computed an ordinary least squares (Additional file 3) and robust linear regression to determine the best fit (Figure 1) [32]. The robust linear regression provided a larger detection range and was used to compute absolute quantification for the assembled transcripts. In total, we computed 9,516 transcripts expressed above 0 molecules per embryo and 8,154 transcripts expressed above 100 molecules per embryo (Figure 4). When restricting the set of transcripts to those with at least 1 BLAST hit against nr, we observed 6,169 transcripts expressed above 100 molecules per embryo (Table 3).

### Blast2GO analysis reveals genes involved in gene regulation

**Figure 5:**
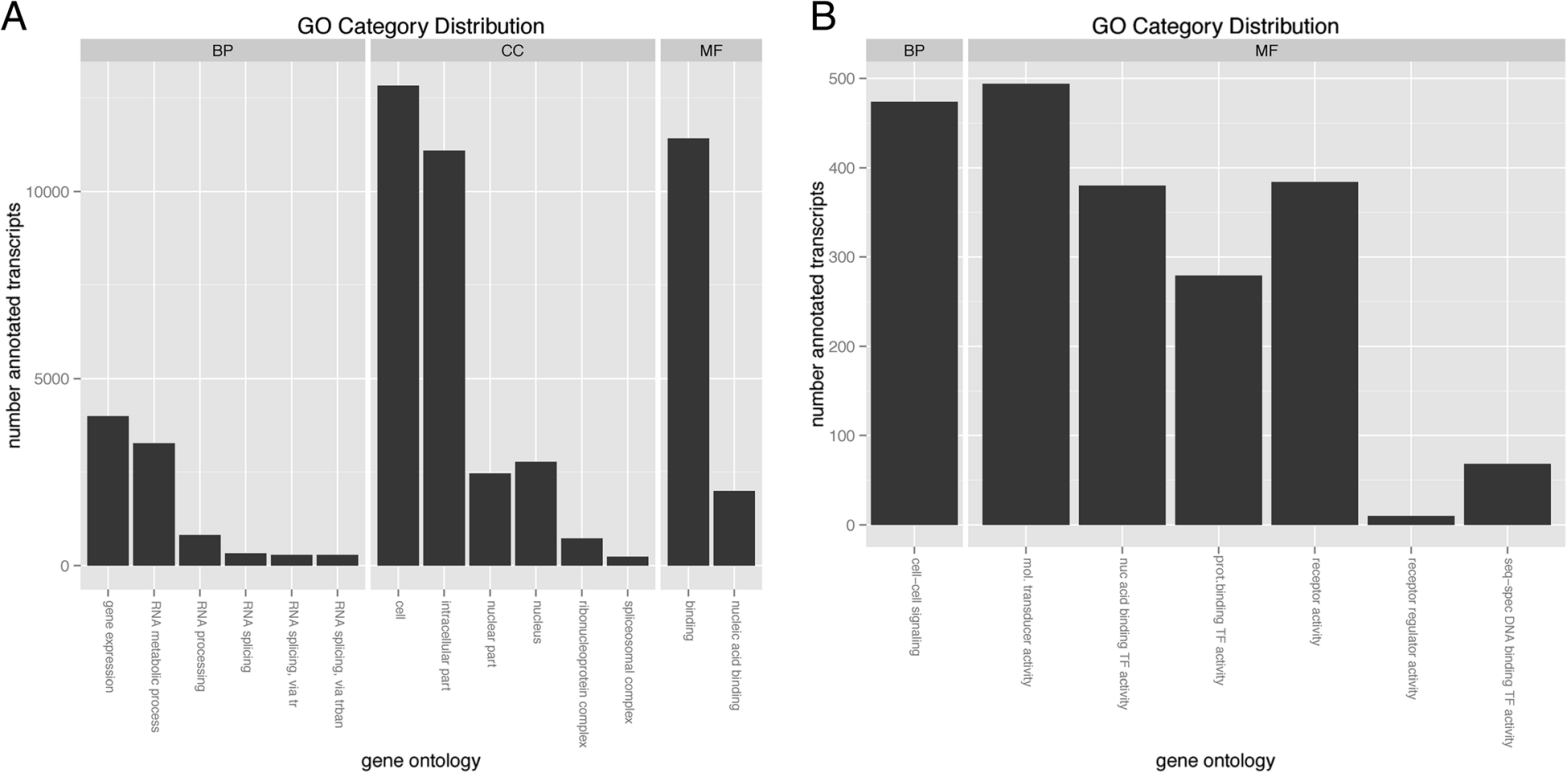
**Gene ontology (GO) analysis: GO term category distribution.** (**A**) Transcripts were processed with Blast2GO and the number of annotated transcripts in each of the selected GO categories is shown for biological process (BP), cellular component (CC), and molecular function (MF). (**B**) A closer look at genes likely important for gene regulation in the categories of cell-cell signaling, molecular transducer, nucleic acid binding activity (transcription factors), protein binding activity (transcription factors), signaling receptors, receptor regulators, and sequence-specific DNA binding transcription factors.

We used Blast2GO to quantify how many transcripts from the Trinity transcriptome fell into well defined gene ontology (GO) categories over all time periods sampled [39]. A sample of the top overexpressed GO terms computed using Fisher’s exact test from the topGO package version 2.10.0 for R [40] are visualized in Figure 5A. GO terms belong to a top level designation of biological process (BP), cellular component (CC), or molecular function (MF) where the titles of the GO terms are in reference to the top level designation; for example, ‘nucleus’ refers to the location of the gene product in the nucleus while ‘gene expression’ refers to a gene product involved in the process of converting gene sequence into RNA or proteins. Definitions for all of the GO terms in Figure 5 can be found in Additional file 3. In order to understand how many transcripts are potentially a part of the embryonic gene regulatory control system, we focused on terms enriched for transcription factors and signaling pathway components (Figure 5B). Nearly 1,000 transcripts combined fell into the 2 transcription factor categories while nearly 1,500 transcripts combined to make up signaling molecules, their receptors, modulators and transducers. Taken together, these 2,500 transcripts provide an estimate of the number of regulatory factors (transcription factors, ligands, receptors, modulators and transducers) present in the *Nematostella* developmental gene regulatory network.

### Transcript family analysis

The transcripts inferred by the Trinity assembler were tested for sequence similarity with known genes. Specifically, the sequence of each transcript was translated and BLASTx was run using the NCBI non-redundant RefSeq database of protein sequences (nr). We combined sequences sharing at least 1 BLAST result with an e-value <5e-5 and a percent identity >80% into a transcript family. The e-value threshold filtered out low confidence BLAST results, while the percent identity filter requires a large percentage of the transcript to match. We systematically tested multiple identity thresholds and found that 80% sequence match yielded a realistic transcript family distribution. Given the natural tradeoff between identification of transcript variants versus paralogous genes, overly stringent requirements of similarity result in an underrepresentation of true homologous relationships; conversely, when similarity thresholds are set too low, distinct transcripts are erroneously grouped together. Because the assembled *Nematostella* transcripts are also included in nr, the sequence similarity threshold is required to be set high. The transcript families therefore represent a mixture of fully assembled gene transcripts, pieces of transcripts, paralogs, or multiple splice forms, entirely compatible with our overarching goals. For BLAST analysis with sequence databases of very different sizes (as in our case, nr vs *Nematostella)* e-values are not an appropriate measure of similarity; in this case a similarity threshold is more informative. Thus, at a similarity threshold of 80% we were able to annotate 48,235 transcripts from the nr database, compared to only 47,193 transcripts when using the *Nematostella* genome alone (Table 3). To do transcript family analysis we used the BLAST hits from the nr database. We inferred 13,293 total transcript families from the 61,835 assembled transcripts with at least 1 BLAST hit. When restricting the transcript family analysis to transcripts expressed over 100 molecules per embryo (MPE) and at least 1 BLAST hit (MPE >100, ≥1 BLAST hit) across the 5 timepoints, we observed a total of 4,055 transcript families from the 6,169 transcripts passing the filter (Table 3). These computations likely represents an underestimate of the true number of genes expressed due to an inability to assemble very lowly expressed transcripts and, in a few cases, grouping paralogous genes together.

**Figure 6:**
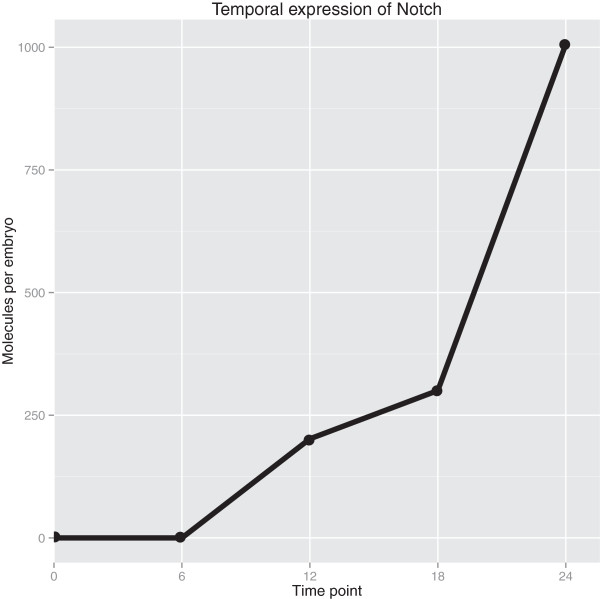
**Temporal quantitative expression of Notch.** Notch transcript family expression over time. Transcripts that were found to be similar to Notch via Basic Local Alignment Search Tool (BLAST) were grouped into a transcript family (three transcripts total, one very short and lowly expressed and the two others nearly identical). The family’s summed expression in molecules per embryo is shown for all five sampled timepoints.

As an example of using the quantitative transcriptome for transcript family analysis, we located the transcripts corresponding to Notch as identified by BLAST. There were three transcripts in this transcript family, one of which is too short and too lowly expressed to be relevant, while the other two are nearly identical and apparently full length. As shown in Figure 6, total Notch molecules per embryo increased from virtually zero copies at 0 h and 6 h, reflecting little or no maternal and early zygotic expression, to significant levels by 12 h, and thence to 1,000 copies at 24 h. A previous study has shown that Notch protein can be seen by *in situ* as early as 20 h, however, that same study only detected Delta-Notch signaling pathway function at later developmental stages [41]. We also see low levels of a putative Delta-like ligand, though we cannot conclude whether it is expressed at functional levels, nor can we make the simplistic conclusion that because both ligand and receptor are present the signaling pathway is functional. Rather our data suggest further investigation is merited, as also stated in Röttinger *et al*. [42], the most systematic study of early *Nematostella* endomesoderm specification to date.

An estimation of the percentage of the genome transcribed during these time periods was computed by taking the length of the longest transcript in each transcript family and dividing that by the length of the genome taken from the estimate in the *Nematostella* genome paper (450 Mbp) [21]. An important caveat is that this is likely an underestimate because some transcripts are not full length. The percent of the genome transcribed above 100 molecules per embryo according to this calculation is 0.368%. The average transcript length for all assembled transcripts is 622.53 bp. When only taking transcripts expressed after spike-in control correction above 0 molecules per embryo, the average transcript length is 456.18 bp. By timepoint, the average of assembled and expressed transcripts is: (0 h) 479.95 bp, (6 h) 487.82 bp, (12 h) 490.54 bp, (18 h) 494.03 bp, and (24 h) 441.29 bp.

## Discussion

The goals of the project discussed in this paper were (1) to identify all the protein-coding genes expressed during the first 24 h of *Nematostella vectensis* development and in so doing (2) to detail a modern, cost-effective, efficient and quantitative series of experimental and computational methods that together make up a transcriptome pipeline for non-model organisms. A key component of our pipeline is the inclusion of NIST RNA spike-in standards for quantification. This allows us to get around the problem of normalizing data to estimate gene expression levels [43], and provides an absolute measure of transcript abundance per embryo.

Many evolutionary developmental biology ‘evo-devo’ research projects have revealed candidate genes in non-model organisms leading to intriguing hypotheses regarding the conservation, or conversely, invention of pathways controlling development [5,44,45]. However, to answer the questions these hypotheses have generated, it is not only the gene homology, presence, absence or spatial localization that needs to be known. To say that a developmental program or subcircuit has been conserved or evolved in a specific way, the *cis*-regulatory network connections between all the regulatory genes involved must be at a minimum known and validated. Candidate genes identified from BLAST analysis will typically only make up a small fraction of the regulatory genes in any pathway. With the advent of next-generation sequencing platforms, identifying all the protein coding genes expressed at any given time during embryonic development is now within the reach of any model system where embryos can be acquired. The lack of a sequenced, annotated genome is no longer a major setback to GRN analysis.

The use of polyadenylated spike-in RNAs provides quantitative information on the absolute abundance of transcripts per embryo. It is important to note the difference between this method of standardization and normalization approaches. The ERCC spike-ins allow us to build a standard curve, in our case a 92-point standard curve. As the quantities of the spike-ins are known, this allows us to infer from the standard curve absolute quantities. Note that since spike-ins are added at the beginning of the library preparation procedures, any variation in preparation efficiencies (that is, technical noise) is in theory accounted for by the spike-ins. Thus, even without absolute quantitation, the use of spike-ins allows direct comparison between samples without the distorting effects of normalization to minimize the effects of technical variation. Further, quantitation by spike-ins also allows us to know the limits of our ability to detect and quantify lowly expressed transcripts. Since low expressed transcripts account for many of the problems in bioinfomatics analysis, our 100 molecules per embryo cut-off allows us to focus our analysis on those transcripts expressed at biologically relevant levels which are also within the linear range of our standard curve. Increasing the sequencing depth and being less conservative with mapping stringency could improve our ability to quantify these lowly expressed transcripts.

**Figure 7:**
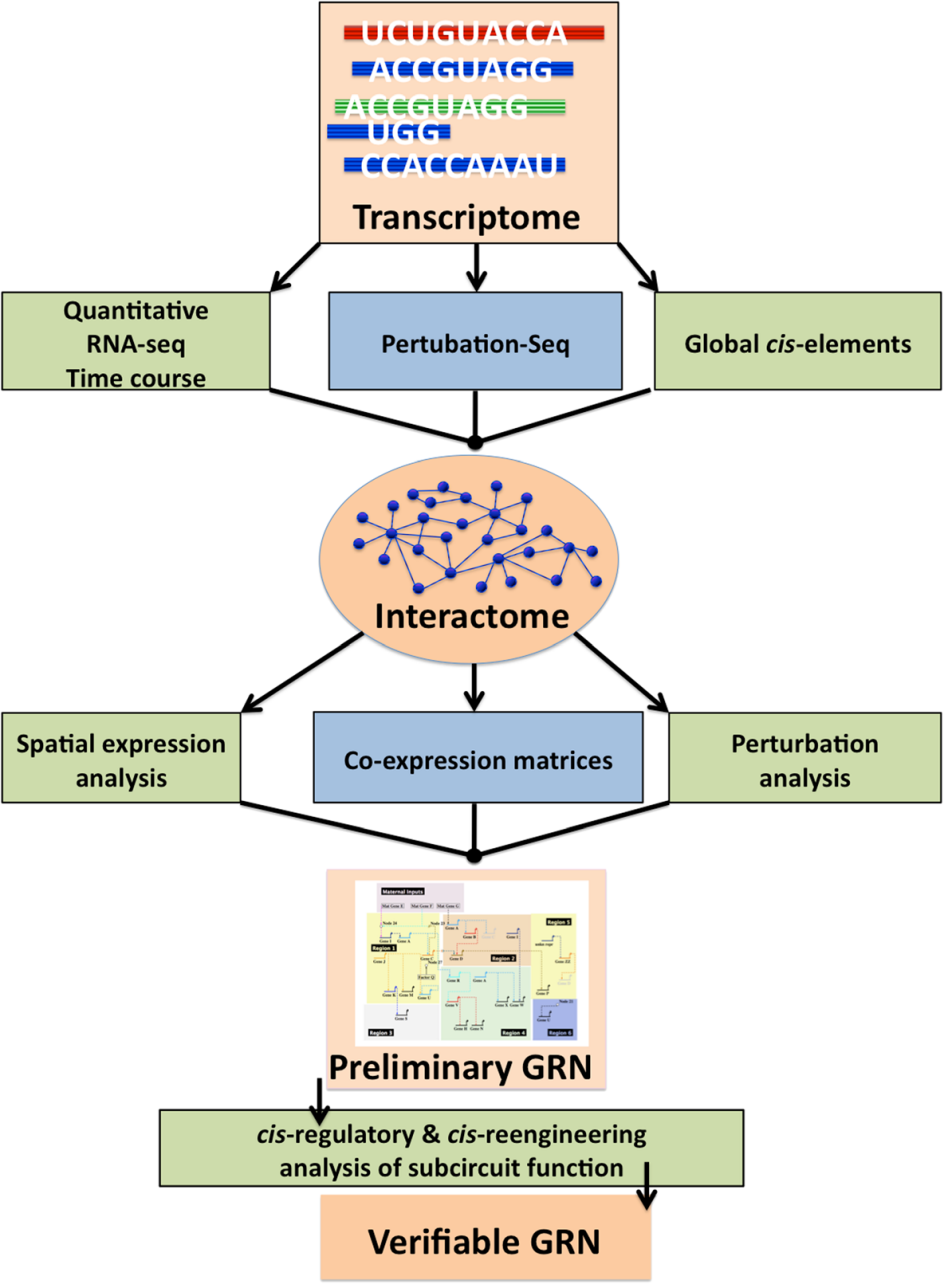
**Gene regulatory network (GRN) construction pipeline flowchart.** A visualization of how an embryonic transcriptome fits into the workflow for constructing a validated GRN. The transcriptome forms the foundation of the GRN because it represents all the transcripts present in the scope of the network. The next immediate datasets to produce are represented at the second level: a high-density, quantitative RNA-seq timecourse which will be mapped to the full transcriptome, for the purpose of high resolution covariance analysis, a ‘Perturbation-seq’ where RNA-seq is used on embryos treated with drugs to major signaling pathways, and a global sequencing effort for *cis-*regulatory elements using either FAIRE-seq or DNase hypersensitivity. Computational analysis of these datasets will produce an interactome with important nodes highlighted. More sensitive investigation of spatial expression, coexpression and perturbation expression (after morpholino treatment) will take the interactome to the level of a preliminary GRN. Finally, *cis-*regulatory analysis of subcircuit function will produce a verified GRN.

This transcriptome pipeline is part of a larger GRN construction pipeline that we are in the process of defining empirically. A visualization of the proposed workflow for constructing a GRN starting from a sequenced and assembled transcriptome is shown in Figure 7. The transcriptome is the starting point and foundation of the GRN because it represents all the transcripts present in the scope of the network. The next datasets to be produced are: a high-density, quantitative RNA-seq timecourse which will be mapped to the full transcriptome, for the purpose of high resolution covariance analysis; a ‘Perturbation-seq’ dataset where RNA-seq is used on embryos treated with drugs against components of major signaling pathways; and a genome-wide sequencing-based search for *cis-*regulatory elements using either FAIRE-seq [46] or DNase I hypersensitivity. A custom computational comparison of these datasets will produce an interactome with clusters representing transcripts that change together from timepoint to timepoint or after a perturbation. More sensitive investigation of spatial expression, coexpression and perturbation expression (after morpholino treatment) will take the interactome to the level of a preliminary GRN. Finally, *cis-*regulatory analysis using bacterial artificial chromosome (BAC) recombination to evaluate subcircuit function will produce a verified GRN with predictive power.

## Conclusions

The embryo of the sea anemone *Nematostella vectensis* provides an important evo-devo model for understanding early animal development, particularly in relation to the question of how initial patterns of differential gene expression emerge along orthogonal body axes. Given *Nematostella*’s position among cnidarians and the molecular evidence thus far, it is possible that a bilaterally-symmetric pattern formation network stretches back to before the Cambrian to a time preceding the Cnidaria-Bilateria bifurcation. However, to make this argument we need a mechanistic understanding of early development in both cnidarian and canonical bilaterian models systems. Moreover, in light of the compare-and-contrast nature of these studies, we need to move away from a candidate gene approach as such methods clearly bias towards the ‘discovery’ of similarities as opposed to differences between regulatory networks. With the advent of genomics, we can now attempt exhaustive *de novo* approaches to define regulatory networks, though challenges in handling RNA-seq data sets still exist. In this report, we have undertaken preliminary steps in defining the *Nematostella* gene regulatory network for early pattern formation by building a comprehensive model of gene expression through 24 h of development. This quantitative reference transcriptome will help us identify, in a minimally biased manner, the most relevant genes to the pattern formation control system. The regulatory network for pattern formation in *Nematostella* will in turn provide a powerful basis for comparison with early development networks from canonical bilaterians.

In summary, we have presented our quantitative reference transcriptome for *Nematostella vectensis* early embryogenesis, which is available on the Woods Hole Data Archive at http://hdl.handle.net/1912/5613 [DOI:http://10.1575/1912/5613]. Additionally, our *de novo* transcriptome pipeline, based on the Trinity assembler, has been designed to meet the needs of the evo-devo community.

## Abbreviations

ERCC: External RNA Controls Consortium; FPKM: fragments per kilobase of exon per million fragments mapped; FSW: filtered sea water; GRN: gene regulatory network; N50 GO: gene ontology; NGS: next generation sequencing; NIST: National Institute of Standards and Technology; nr: non-redundant BLAST database; (q)PCR: (quantitative) polymerase chain reaction.

## Competing interests

The authors declare they have no competing interests.

## Authors’ contributions

ST and JS designed the experiment; DA and SI, in consultation with ST and JS, designed the computational pipeline for the *de novo* transcriptome assembly. ST performed the experiments. DA performed the Trinity assembly and computational analysis. ST performed the digital normalization and Oases assembly. All authors contributed to the writing of the manuscript. All authors read and approved the final manuscript.

## Supplementary Material

Additional file 1 diginorm_velvet_oases_commands.txt. Click here for file

Additional file 2 qc_trinity_commands.txt. Click here for file

Additional file 3 Ordinary least square regression plots. Click here for file

Additional file 4 Gene ontology (GO) term definitions. Click here for file
