# Supplementary figures and images for: A quantitative reference transcriptome for Nematostella vectensis early embryonic development: a pipeline for de novo assembly in emerging model systems
Source: EvoDevo. 2013 Jun 3;4:16. doi: 10.1186/2041-9139-4-16 (PMC3748831; doi:10.1186/2041-9139-4-16)

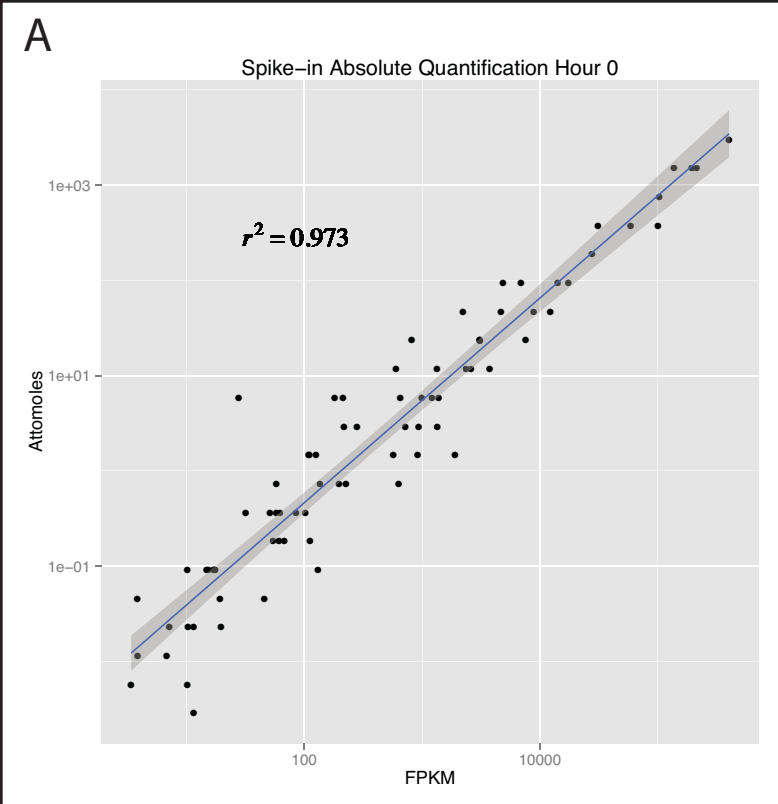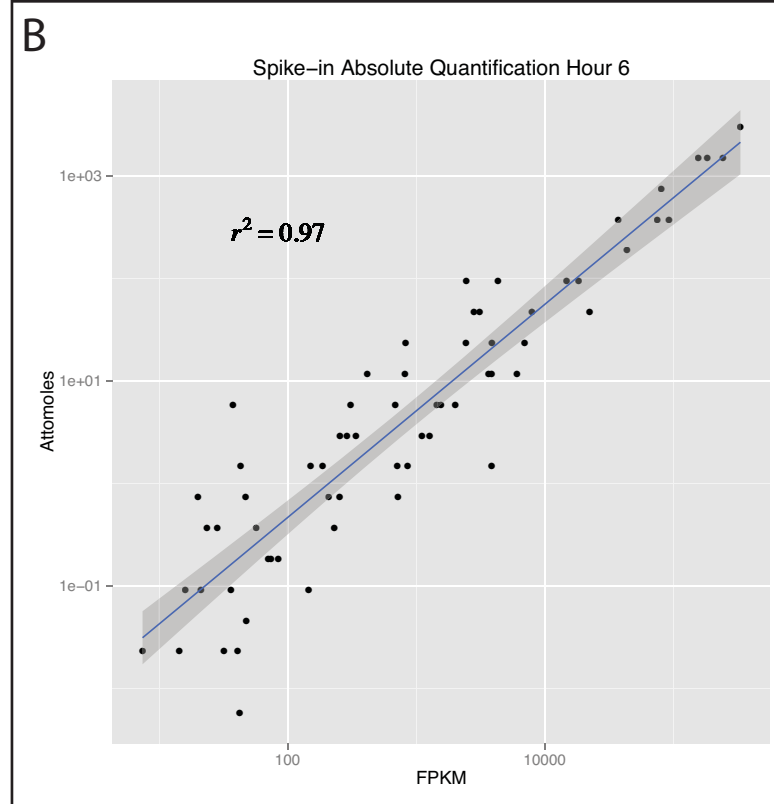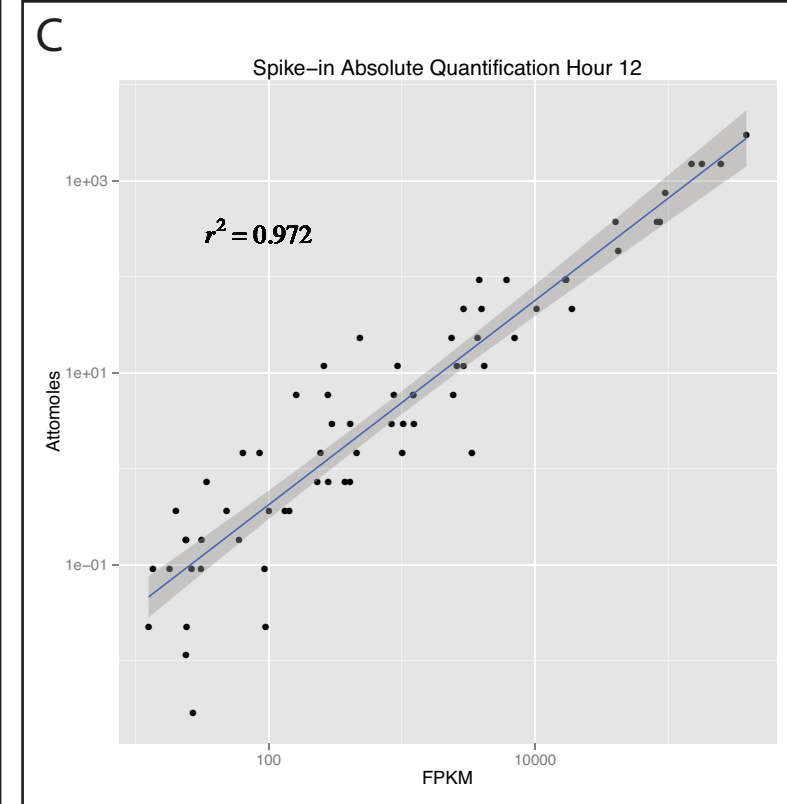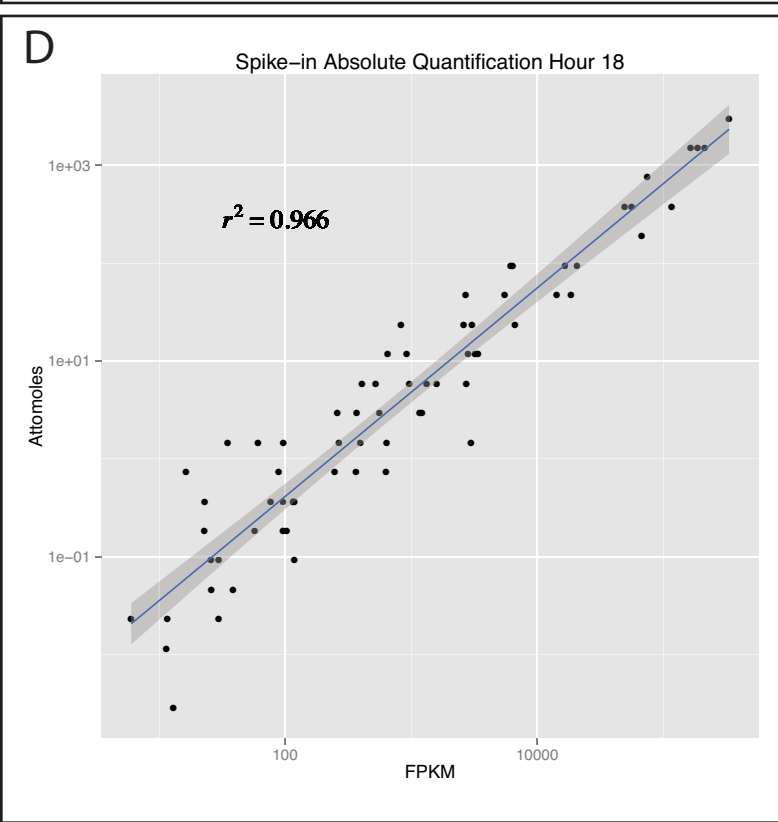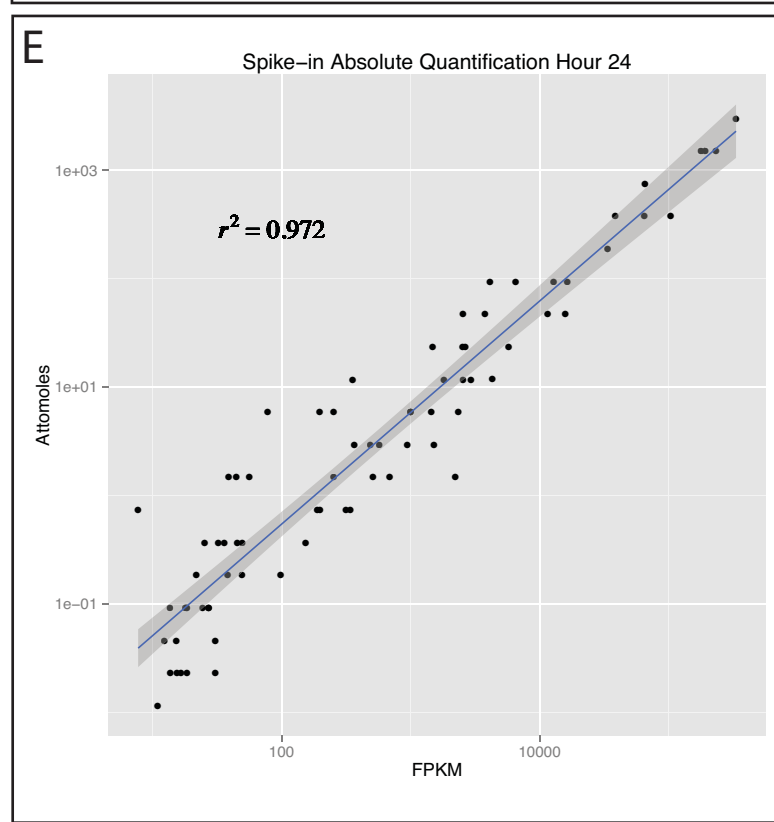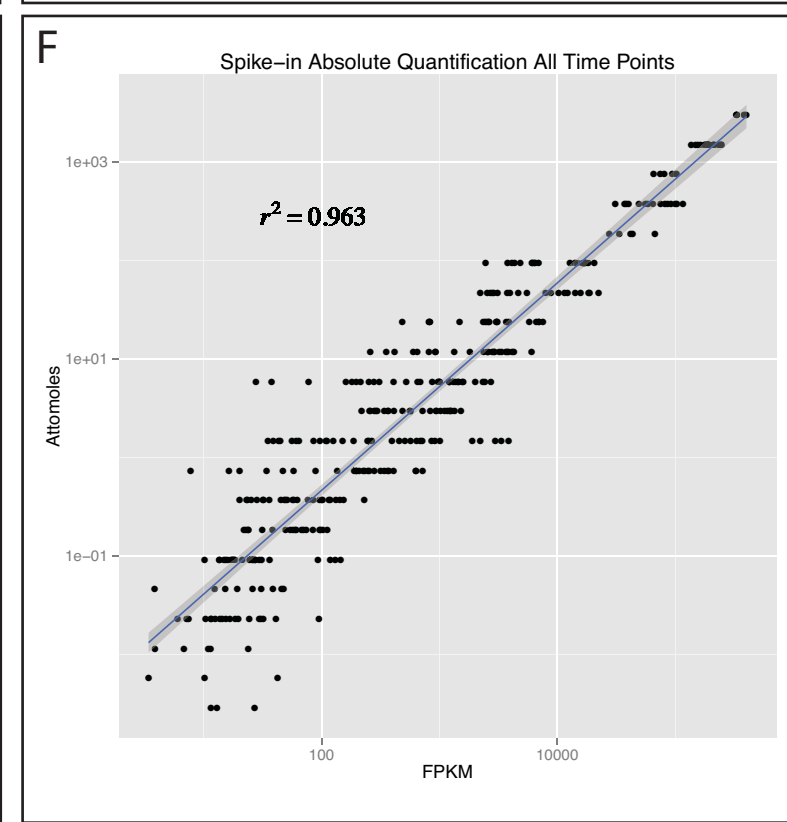

Supplement: Additional file 3 — Ordinary least square regression plots. [file 2041-9139-4-16-S3.pdf]
